# Hydrogels based on poly(vinyl alcohol) for cartilage substitution

**DOI:** 10.1080/07853890.2021.1896896

**Published:** 2021-09-28

**Authors:** A. S. Oliveira, R. Colaço, A. P. Serro

**Affiliations:** a CQE, IST – Universidade de Lisboa, Lisboa, Portugal; b IDMEC, IST – Universidade de Lisboa, Lisboa, Portugal; c Centro de Investigação Interdisciplinar Egas Moniz (CiiEM), Egas Moniz Cooperativa de Ensino Superior, Caparica, Portugal

## Abstract

**Introduction:**

Cartilage damage is an important concern because of the tissue's limited ability to repair itself [1,2]. Among the existing strategies to replace cartilage, hydrogels have been widely considered due to their ability to easily mimic the natural tissue [1,2]. Poly(vinyl alcohol) (PVA) based hydrogels have been the focus of a large number of studies as they are easy to produce, have excellent biocompatibility, low toxicity, high water content, and stability [2]. The aim of this study was to evaluate the effect of different preparation conditions of PVA hydrogels on the properties of the materials.

**Materials and methods:**

PVA (MW 145000 Da) aqueous solutions (15% w/v) were prepared at 95 °C for 6 h, poured into flat moulds and cooled down to room temperature. Cast drying samples (CD_4_ and CD_30_) samples were dried at 4 and 30 °C until they reach a constant weight. Freeze-thawing samples (FT_0_ and FT_NaCl_) were prepared with 5 cycles of 10 h at −20 °C and 2 h at room temperature. For FT_NaCl_ samples, an aqueous solution of NaCl (25% w/w) was pre-added to the initial polymer solution in a proportion of 1:10 (v/v) relative to PVA. The hydrogels were characterised concerning equilibrium water content (EWC), surface morphology (analysed by Scanning Electron Microscopy (SEM, JEOL JSM-7001F)), thermotropic behaviour (studied by differential scanning calorimeter (NETZSCH 200 F3 Maia) with thermograms being recorded over the range of 20–280 °C at a heating rate of 10 °C/min), mechanical performance (evaluated by uniaxial tensile and unconfined compression tests carried out in a TA.XT Express Texture Analyzer) and friction behaviour (pin-on-disc tests done in linear reciprocal oscillation mode using stainless steel 316 L balls as counter-bodies, with loads of 10 and 20 N, in phosphate buffered saline (PBS) solution and simulated synovial fluid (SSF) solution with hyaluronic acid and bovine serum albumin).

**Results:**

CD_4_ and CD_30_ showed an EWC in the range of 63–66% while that of FT_0_ and FT_NaCl_ varied between 87–89%. SEM micrographs revealed different structures for CD and FT samples. The surface of CD gels was smoother with no evident porosity, while FT materials exhibited holes in different porosity patterns. FT_NaCl_ presented smaller pores than FT_0_, although a minor number of large pores coexist. The degree of crystallinity (35–36%), glass transition temperature (43–44 °C), and melting transition temperature (214–216 °C) were similar for all samples. Regarding mechanical performance, the elastic modulus in both compression and tensile, was inferior for the FT materials, in particular for FT_NaCl_. In tensile experiments, CD gels had the highest ultimate tensile strength and toughness. Concerning tribology behaviour, the friction coefficients (CoF) of all samples were low and similar in PBS solution when 10 N of force was applied. For 20 N, the CoF values of the CD materials decreased. In SSF solution, CD samples presented higher CoF with both tested forces.

**Discussion and conclusions:**

The properties of the PVA hydrogels were strongly influenced by the preparation conditions. CD method proved to be more suitable for producing PVA cartilage substitutes. It led to more compact, rigid, and resistant materials with adequate values of EWC and CoF.
